# Event and Comment

**Published:** 1910-05-15

**Authors:** 


### DENTAL REGISTER.

Vol. LXIV. May 15, 1910. No. 5.

### EVENT AND COMMENT.

### WILL THE GOLD FILLING COME BACK?

Since the beginning of the "New Departure" propaganda, the first article of its creed being, "In proportion as teeth need saving, gold is the worst material to use," down through oxyphosphate cements, porcelain inlays, gold inlays and silicious cements to the present time, those, who by strenuous effort mastered the technical details of gold manipulation have jealously guarded its reputation as the ideal and standard filling material. It is undoubtedly true that gold as a filling material is the most exacting of all materials from the manipulative standpoint and naturally those who have paid the cost and learned her secrets will naturally maintain loyalty and be suspicious and even jealous of any new comer that threatens to dethrone their idol. Gold has so long been the standard of comparison at least, that we note a renewed tendency to discuss methods of cavity preparation and manipulative details, as though there was a disappointment in the promised substitutes above mentioned, or that there was something more to be said about technical methods. Have we made no advancement or gained no advantage from the testing out of the several new materials presented during the past decade of experimentation?

If we turn back to the time of Marshall H. Webb, and note the wonderful contour gold foil operations he made with such consummate skill and art, and note also the tremendous impression he made on the profession in stimulating it to greater achievements in the skillful manipulation of gold foil we can easily decide that little further development has taken place along that line. In fact we have probably degenerated from his high standard, at least we do not now restore the entire crowns of molar teeth with gold foil at great cost of money, time and the health of both patient and operator. Without intending to detract at all from the magnificent efforts of this remarkable genius we now know and practice better methods and have learned that gold foil has its place in dental art, and that while it may still be the king of filling materials, it must divide honors with other materials and methods where they meet all the requirements more completelv. The present ease and perfection with which the gold inlay is constructed has given gold foil filling its hardest knock. The porcelain inlay has made its place secure, and the silicious cements are meeting a demand in a promising manner, but their trial is still on. In the mean while we have not discarded our gold pluggers, although in many good practices they lie idle or undisturbed for days at times. So far as we can now see gold foil fillings will never come back to their former universal employment; but because of certain inherent qualities in the material itself and the possibilities of definite and practicable manipulation there can be little doubt that when the excitement of the chase after the new has spent itself and we have found that all things have limitations we shall be glad to get back again to a material that has much to satisfy and comparatively few limitations. It would therefore seem to be the wiser course for us to hold on to the tried and satisfactory gold foil filling until we have had time to make comparison with the newer materials before placing our reputations on a basis that may bring disappointments and possible censure. When in doubt give the option to that material which the largest experience has proven most dependable.

